# Investigating Performance of an Embedded Machine Learning Solution for Classifying Postural Behaviors †

**DOI:** 10.3390/s25144262

**Published:** 2025-07-09

**Authors:** Bruno Andò, Salvatore Baglio, Mattia Manenti, Valeria Finocchiaro, Vincenzo Marletta, Sreeraman Rajan, Ebrahim Ali Nehary, Valeria Dibilio, Mario Zappia, Giovanni Mostile

**Affiliations:** 1 Department of Electrical, Electronic and Computer Engineering (DIEEI), University of Catania, 95123 Catania, Italy; salvatore.baglio@unict.it (S.B.); mattia.manenti@phd.unict.it (M.M.); valeapp98@gmail.com (V.F.); 2 STMicroelectronics S.r.l., 95121 Catania, Italy; vincenzo.marletta@gmail.com; 3 Department of Systems and Computer Engineering, Carleton University, Ottawa, ON K1S 5B6, Canada; sreeramanr@sce.carleton.ca (S.R.); ebrahimali@cmail.carleton.ca (E.A.N.); 4 Department of Medical, Surgical Sciences and Advanced Technologies “G.F. Ingrassia” (DGFI), University of Catania, 95123 Catania, Italy; dibilio83@hotmail.it (V.D.); m.zappia@unict.it (M.Z.); g.mostile@unict.it (G.M.); 5 Oasi Research Institute—IRCCS, 94018 Troina, Italy

**Keywords:** postural sway classification, inertial sensor, multi-layer perceptron, experimental assessment, noise robustness

## Abstract

Postural instability is one of the main critical aspects to be monitored in the case of degenerative diseases, and is also a predictor of potential falls. This paper presents a multi-layer perceptron approach for the classification of four different classes of postural behaviors that is implemented by an embedded sensing architecture. The robustness of the methodology against noisy data and the effects of using different sets of classification features have been investigated. In the case of noisy input data, a reliability index of almost 100% has been obtained, with a negligible drop (less than 5%) being shown for the whole range of noise levels that was investigated. Such an achievement substantiates the better robustness of this approach with respect to threshold-based algorithms, which have been also considered for the sake of comparison.

## 1. Introduction

Wearable devices equipped with motion sensors have been widely proposed for monitoring frail individuals, particularly in relation to mobility and fall risks [1,2,3]. Reduced mobility and instability are key clinical aspects of degenerative disorders such as Parkinson’s disease (PD), where multiple factors compromise patients’ health and well-being [4].

In patients with parkinsonism, standard diagnostic techniques are employed to assess motor impairment, evaluate the treatment response, and support differential diagnosis [5,6]. Recently, a data-driven approach that leverages features extracted from monitoring systems was proposed to clinically characterize individuals with neurological disorders based on their mobility and stability features [7].

Postural stability is typically assessed experimentally by applying perturbations in various directions to identify the primary axes of instability. While healthy individuals can adapt their postural responses to such mechanical disturbances, older adults, particularly frail patients with parkinsonism, are often unable to do so. They tend to present reduced stability margins in all directions, with their backward and lateral sway being especially compromised [8]. Various approaches have been proposed to study postural sway dynamics in controlled environments, such as hospitals or healthcare facilities. These methods enable reliable and well-referenced analyses by leveraging standard equipment, such as force platforms and vision-based systems [9,10]. The work presented in [9] reports on the main procedures aimed at investigating postural stability, which include analyzing dynamics of the center of pressure (CoP) and the use of several types of equipment, including force plates and balance masters. Although more complex, vision-based approaches have also been widely reported in the literature, ranging from professional systems to low-cost alternatives [11,12], these methods enable the reliable estimation of CoP dynamics, which can be correlated with the severity of the instability being investigated.

The above-mentioned methods, although very accurate, suffer from the need to perform tests with end-users in structured environments. Hence, these systems are not suitable for daily monitoring that is accomplished at home or in other environments that are attended every day. This highlights the necessity to develop efficient and reliable wearable solutions that are capable of continuously monitoring patients and detecting anomalies or deviations from typical behaviors. This approach is also strategically useful for the implementation of fall prevention strategies.

Wearable sensing devices can be used to estimate instrumental features of instability among frail people [13], which are also related to specific directions that show an altered postural response. The data-driven analysis of such features could then provide strategic clinical information to differentiate stable from unstable patients and the correlated risks of falling.

As further discussed in Section 2, notable results are already present in the state of the art, with different solutions implementing machine learning methods in the field of postural analysis. However, there is a lack of research specifically focused on classifying distinct postural sway behaviors. High reliability and robustness, even in case of noisy data, are mandatory characteristics required by these solutions. Furthermore, with the aim of accomplishing the real-time monitoring of postural sway, machine learning classifiers must be deployable in low-cost embedded systems.

In line with the above statements, the 4FRAILTY project, focused on the development of an integrated infrastructure, is aimed at improving the quality of life of frail users living in their own environments. One of the challenges addressed by the project is the development of wearable solutions that allow for the monitoring of postural behaviors and the classification among different classes of postural sways, with regards to patients with PD. It must be underlined that this task is different than simply distinguishing stable and unstable behaviors [14] and more challenging with respect to posture/activity detection and recognition [13].

This article is a revised and expanded version of the paper [15] entitled “Machine Learning approach to classify Postural sway Instabilities”, which was presented at the 2023 IEEE International Instrumentation and Measurement Technology Conference (I2MTC) in Kuala Lumpur, Malaysia, during 22–25 May 2023. In [15], as a preliminary study, the development of a multi-layer perceptron (MLP) approach for the classification of four different classes of postural sway behaviors, exploiting an embedded sensing architecture, is discussed.

In this work, the approach presented in [15] is further addressed. The main contribution of this work is related to the investigation of the robustness of the developed methodology for postural sway classification against noisy data. This task is crucial, since artifacts can arise from the real use of this system. The results demonstrate that the MLP approach is more robust in terms of accuracy and reliability with respect to the threshold-based algorithm investigated in [16]. Furthermore, as will be shown, training the MLP model with a noisy dataset can improve its performance in terms of accuracy, with a slight decrease in reliability. As a further outcome, the effect of using different sets of classification features is addressed. Feature selection is important for providing the classifier with the correct amount of information, as irrelevant or redundant features could reduce the model’s accuracy. Also, selecting a suitable number of features is important to prevent overfitting (in case too many features are used) and to reduce the computational demand during both feature extraction and inference. Results obtained through this analysis confirmed that the number of features could be reduced from four to three, which decreases the computational requirements without compromising both the accuracy and reliability of the methodology. Furthermore, the deployment of the MLP algorithm in an embedded architecture is considered, along with a dedicated assessment session.

The main novelties introduced by this work, with respect to [15], can be summarized as follows:

– The demonstration of better performances of the proposed machine learning classification methodology compared to threshold-based algorithms [16], especially when noisy data can affect the inference phase;
– The comparison of the two approaches, in cases where different sets of features are used to feed the classification algorithms;
– The investigation of performances shown by the MLP classification approach, in the case that a noisy dataset is used during the training phase;
– The analysis of outcomes achieved by deploying the machine learning model in the embedded system.

In the following, the main outcomes of the proposed embedded solution, with respect to other approaches available in the literature, are listed:

– The machine learning-based methodology allows the classification of four different postural dynamics, showing better performance in terms of accuracy and reliability with respect to threshold-based solutions [16];
– The system allows for monitoring postural behavior both in indoor and outdoor environments;
– The system does not require structured environments, such as dedicated laboratories and the related setup, nor specialized operators;
– The adopted approach shows an intrinsic robustness against the absolute positioning of the node, thanks to the adoption of features which use relative distances between the node and the floor/belt joints. This is an advantage with respect to solutions that exploit the raw time evolution of signals provided by inertial measurement units (IMUs);
– Ease of implementation of the classification approach in embedded systems, thanks to dedicated libraries that allow the deployment of machine learning models.

The rest of this work is structured as follows. Section 2 reports an overview of the state of the art, highlighting the added value of this work with respect to related works. Section 3 includes the presentation of the experimental setup and the proposed methodology. The performance of the postural sway classification approach is discussed in Section 4. In Section 5, the implementation of the machine learning algorithm in the adopted embedded architecture is presented, along with the results of a dedicated assessment session. Finally, Section 6 is dedicated to the conclusions and discussion about future developments.

## 2. Related Works

Different contributions in the literature address wearable sensing nodes and the use of features to implement postural monitoring tasks [17]. Most solutions make use of IMUs to extract information from users’ movements and characterize their postural dynamics [18]. The tasks performed by these systems are highly varied, addressing several types of posture analysis. In the following, examples of threshold-based and machine learning approaches are reported.

### 2.1. Threshold-Based Approaches

The validity and sensitivity of an inertial sensor for the assessment of postural sway is addressed in [19]. In particular, the work reports the results of laboratory tests in which the performances of inertial sensors, force plates, and rigid-body kinematics across traditional balance tests are compared. The characterization of motor subtypes of patients with PD, enabling their quantitative classification, is investigated in [20]. The performance analysis of a head-mounted IMU, implementing postural sway measurements, is addressed in [21]. In [22], the ability of a pendant sensor to discriminate between balance conditions is investigated. The use of a smartphone for the monitoring and prediction of falls by measuring postural sway indicators is presented in [23]. In [24], the authors investigated the possibility of adopting a wearable embedded system to simultaneously analyze the three-dimensional angular position of the user in eight points of their body. The analysis of postural kinetics by using IMU, under different postural stress conditions, is discussed in [25], with regards to patients with Alzheimer’s disease. In [26], a threshold-based approach is proposed for the detection of postural instability. The proposed solution exploits information provided by a triaxial accelerometer to reconstruct the stabilogram and to extract features that are useful for discriminating among stable and unstable behaviors. The methodology exploits a set of threshold-based rules, while performance indexes are used to quantify the robustness of the proposed methodology and the reliability of its estimations.

Regarding the exploitation of frequency-based metrics, in [27], the assessment of the user’s postural control strategy is addressed by using the discrete wavelet transform (DWT) theory. The possibility of extracting timescale components from the CoP time series, with the aim of investigating postural control, is discussed in [28]. The use of features obtained by applying DWT theory to accelerometer signals is investigated in [29], with the aim of distinguishing between stable and unstable behaviors.

The main limitation of threshold-based strategies is the difficulty of appropriately defining separation elements, which is what allows the performance of a sharp clustering between different postural behaviors, especially in the presence of noisy data. Such a finding highlights the need to use advanced strategies to implement postural sway classifications. This is particularly relevant in cases where the recognition of different classes is required, as opposed to the task of simply discriminating between stable and unstable behaviors. To this end, machine learning strategies could represent a more effective approach.

### 2.2. Machine Learning-Based Approaches

In the literature, different examples of machine learning approaches for postural analysis are reported. The monitoring of users’ pressure and sitting posture, exploiting decision tree and random forest classifiers, is discussed in [30]. The performance of different machine learning algorithms for the monitoring and recognition of sitting posture, by exploiting a pressure sensor array, is assessed in [31]. Machine learning-based approaches have also been efficiently exploited for the development of fall detection solutions [32,33,34].

A machine learning approach to assess the accuracy and the use of postural sway metrics to differentiate individuals with multiple sclerosis from healthy controls, as a function of physiological fall risk, is presented in [35]. A random forest algorithm has been used to predict individuals’ fall risk grouping. The use of motion information and machine learning strategies to classify postural control stability, according to joint node trajectory patterns, is investigated in [36].

The assessment of postural sway for post-stroke patients, using a machine learning-based clustering method, has been presented in [37]. In [38], a neuro-fuzzy (NF) inference model designed to perform a raw classification among stable and unstable postural dynamics is presented. In [39], a fuzzy logic posture classification algorithm, aimed at assessing if a user adopts good or bad postures for specific time periods, is described. Information related to the CoP and posture adoption time was used to feed the classification algorithm.

In [14], a comparative analysis of different strategies for postural instability detection is presented. Threshold approaches that exploit time-based features are compared to the use of NF inference to exploit time-based and DWT features. The same work investigates the robustness against noise of the different methodologies, highlighting the better performances of NF-based approaches.

A machine learning approach that exploits a MLP to classify four different classes of postural sway behaviors by using data provided by an embedded architecture equipped with a triaxial accelerometer is discussed in [15].

Table 1 summarizes key features of the above-referenced literature to properly contextualize the position of this work with respect to available approaches.

## 3. The Proposed Methodology

The device adopted for the classification of postural behaviors is the embedded multi-sensor board SensorTile.box (STEVAL-MKSBOX1V1) by STMicroelectronics (Geneva, Switzerland). This platform includes an ultra-low-power ARM Cortex M4 MicroController Unit (MCU) and a LIS2DW12 triaxial MEMS accelerometer with 16-bit data output. The latter has selectable full scales of ±2/4/8/16 g and can measure accelerations with output data rates from 1.6 Hz to 1600 Hz. For the addressed task, it has been set to acquire data at a sampling rate of 100 Hz and a full scale of ±2 g.

**Figure 1:**
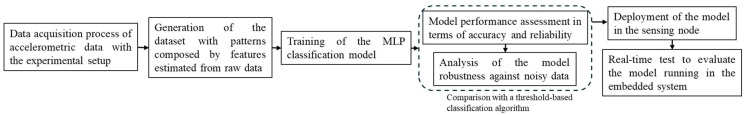
Main steps of the methodology and results presented in this work.

For the sake of convenience, the overall methodology, which is detailed in the next sections, is summarized in Figure 1.

### 3.1. Features and the Dataset Adopted for the Classification Strategy

Assuming that the sensing node is placed in the central chest region of the end-user, the three acceleration components *A_x,y,z_* allow for the estimation of the time evolutions of the antero-posterior (AP) and medio-lateral (ML) displacements by exploiting the following relationships [26]:

(1) $$DAP=H_{1}\cdot \frac{A_{z}}{\sqrt{A_{y}^{2}+A_{x}^{2}}}$$

(2) $$DML=H_{2}\cdot \frac{A_{x}}{\sqrt{A_{y}^{2}+A_{z}^{2}}}$$

where $H_{1}$ is the distance between the sensor node and the floor, while $H_{2}$ is the distance between the node and the belt joint.

**Figure 2:**
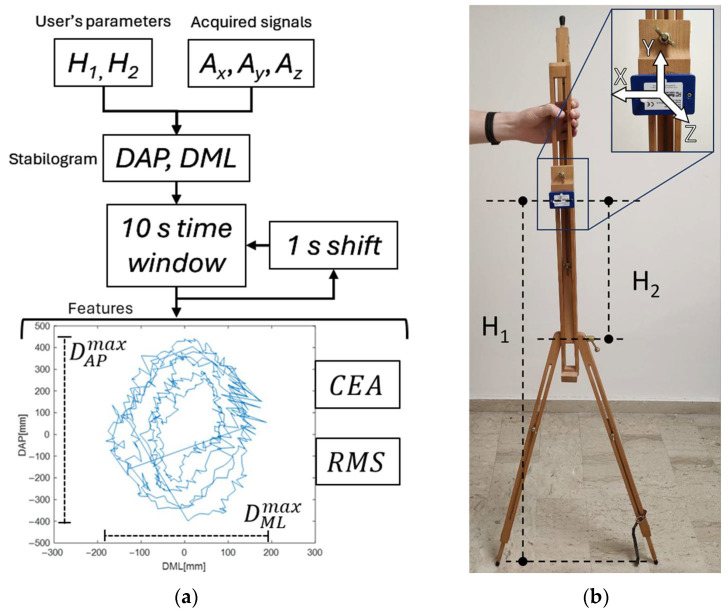
(**a**) Data flow for features generation. Examples of features are reported in the stabilogram; (**b**) the architecture adopted to reproduce postural sway behaviors.

The combination of DAP and DML dynamics generates the stabilogram [40], which allows for the definition of a set of features that are useful in implementing the classification task. In [16], a strategy to select the best features among several quantities has been presented and discussed. According to the results obtained in the work, and as schematized in Figure 2a, the features considered for this study are the following:

– $D_{AP}^{max}$ (m): maximum displacement range in the AP direction;
– $D_{ML}^{max}$ (m): maximum displacement range in the ML direction;
– $CEA=\pi \cdot a\cdot b\;\left(m^{2}\right)$: ellipse area including 95% of the stabilogram plot, where *a* and *b* represent the two semi-axes of the ellipse. $a=CSF\cdot \sigma_{AP}$ (m) and $b=CSF\cdot \sigma_{ML}$ (m). *CSF* is a confidence scaling factor whose value, in the case of the 95% ellipse, is 2.4477. $\sigma_{AP}$ and $\sigma_{ML}$ are the standard deviations of DAP and DML, respectively;
– $RMS=\sqrt{\frac{\sum_{i=1}^{N}d_{p}{\left(i\right)}^{2}}{N}}$ (m): root mean square displacement, where $d_{p}\left(i\right)$ is the distance between two adjacent points of the stabilogram.

To generate a dataset for the implementation of the classification task, the dedicated structure shown in Figure 2b has been used. This setup has been chosen because it allows for miming the following dynamics during the generation of the dataset: standing stable behaviors (STs), AP and ML instability dynamics, and general unstable behaviors (UNSTs).

By using the structure presented in Figure 2, STs have been emulated by maintaining the structure in a standing-still condition. ML displacements have been obtained by tilting the structure around the belt joint in the ML direction, while the AP ones have been generated by tilting around the bottom joint in the AP direction. The UNSTs have been achieved by randomly tilting the structure in different directions.

The choice of using the emulating structure shown in Figure 2 is due to the need to rapidly and safely generate the required dataset, which is not compliant with end-users’ recruitment procedures, as well as with their involvement in experiments. Hence, this study represents a first mandatory step which, of course, requires further efforts for the involvement of end-users, but which allows for model refinement and system validation. While being aware of limitations given from artifacts introduced by the rigid structure with respect to human-body dynamics, the results shown in the next section demonstrate the approach’s robustness against noisy data. Therefore, it is expected that the methodology will keep its validity even when adopted with real users.

The firmware implemented in the MCU allows for running the continuous acquisition of samples generated by the triaxial accelerometer. Such data are collected in time windows of 10 s, as suggested in the literature [14]. Consecutive windows are overlapped by 1 s, which represents a convenient compromise between computational demand and continuous postural sway monitoring, without losing information that is useful in recognizing different kinds of events. This process is represented in Figure 2a.

For each time window, the above-mentioned features are calculated in real-time by the sensor node. The dataset consists of patterns composed by the 4 features and a label which identifies the corresponding type of dynamics (ST, AP, ML, or UNST). After 10 s from the start of the acquisition process, patterns are generated with a rate of 1 s, according to the time window shifting algorithm.

**Figure 3:**
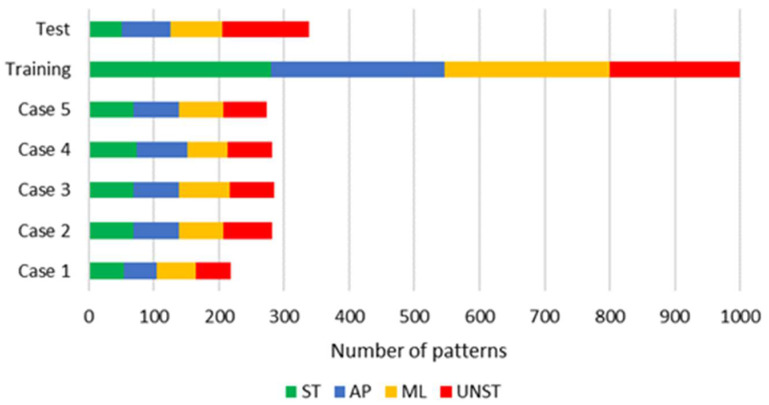
The dataset and its distribution among different cases and training/test subsets. The overall number of patterns for each class is: 331 ST, 340 ML, 334 AP, and 333 UNST.

Details of the dataset composition, for each investigated behavior and considered case, are represented in Figure 3. Patterns have been extracted by 20 records that last between 60 s and 87 s and have been collected in laboratory with standard environmental conditions (i.e., room temperature and pressure, which, anyway, are not expected to affect the system’s behavior). The number of patterns depends on the length of the acquisitions, which is different for each of the classes considered due to practical reasons. Five different cases have been considered, which are characterized by different heights of the sensor node mounted on the structure. This allows for the assessment of the classification methodology’s robustness against different node positions.

The dataset used to perform this study has been generated by randomly dividing the whole set of patterns into training and test subsets (with a 75%/25% split).

### 3.2. The Machine Learning Approach for Postural Sway Classification

The machine learning classification algorithm is based on a MLP architecture implemented by Keras Tensorflow libraries in Python 3.10.0. The choice of this type of artificial neural network, among the wide variety of available machine learning algorithms, is justified by its ease of implementation in the embedded sensing node. For the sake of completeness, in Section 4, a comparison between the performance of the MLP algorithm against that of three other standard machine learning techniques is discussed.

**Figure 4:**
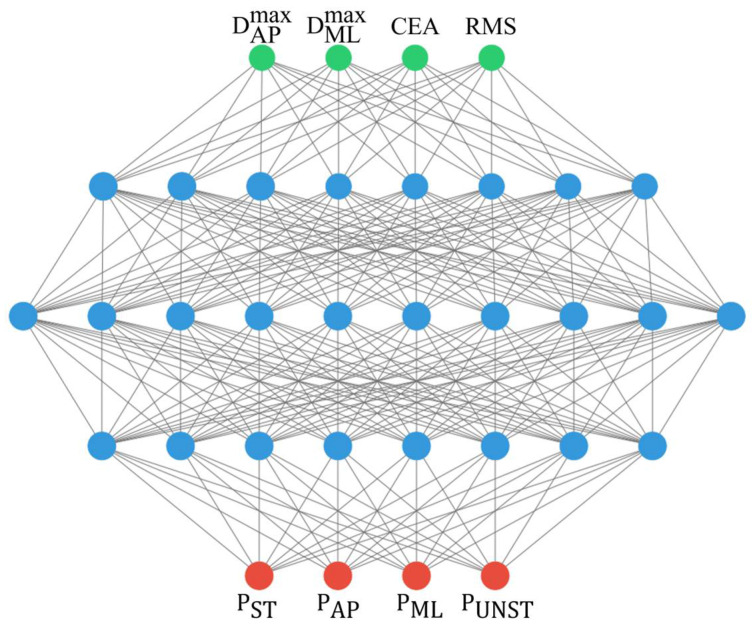
The proposed MLP architecture, in the case where all four features are used as the model input.

The MLP model is composed of one input layer, three hidden layers (with 8, 10, and 8 neurons, respectively) and one output layer, as reported in Figure 4.

The layers are fully connected and ReLU (rectified linear unit) activation functions have been used for the input and hidden layers, while, for the output layer, a “softmax” activation function has been implemented to obtain output values ranging between 0 and 1 (prediction probabilities P). During the training, a batch size of 32 was adopted, exploiting the “Adam gradient descent” optimization algorithm with a logarithmic loss function. The accuracy, used as the evaluation metric during the development phase, is defined as follows [41]:

(3) $$Q\%=100\left[1-\frac{\sum_{i=1}^{N}\gamma_{i}}{N}\right]$$

with

$$\gamma_{i}=\left\{\begin{array}{c} 0\;if\;\left|C_{i}-C_{i}^{exp}\right|=0\\ 1\;if\;\left|C_{i}-C_{i}^{exp}\right|>0 \end{array}\right.$$

In the above equations, the terms are defined as follows:

– N is the number of considered patterns;
– $C_{i}^{exp}$ are the expected classes;
– $C_{i}$ are the classes estimated by the model.

To assess the reliability of the classification task performed by the MLP algorithm, the following reliability index *RI* has been defined:

(4) $$RI=\frac{P_{1}-P_{2}}{P_{1}}$$

where $P_{1}$ and $P_{2}$ are the two highest predicted probabilities.

Index (4) is strategic, especially during the real operation of the system, where the accuracy index (3) cannot be used because the target class $C_{i}^{exp}$ is unknown.

For the estimation of the overall performance of the MLP classification approach, the following indexes have been also computed:

(5) $$RI_{Mean}=mean\left(RI\right)$$

(6) $$RI_{Std}=std\left(RI\right)$$

where $mean\left(\cdot \right)$ and $std\left(\cdot \right)$ are the mean and standard deviation operators, respectively.

## 4. Results and Discussion

Before discussing the new findings of this work, for the sake of convenience, Table 2 reports the main results, which were already presented in [15], including the accuracy and reliability of the model. In particular, the comparison between a threshold-based algorithm [16] and the machine learning approach exploiting the four features described in Section 3.1 is shown. The threshold algorithm uses a set of if-then rules, where each feature is compared to a corresponding optimal threshold estimated by the Receiver Operating Characteristic curves theory [42].

The obtained results show that the MLP algorithm performs better than the threshold approach, with $RI_{Mean}$ index values close to 100% in most of the cases, which contrasts with the 60% obtained for the threshold algorithm. The superior performance of the MLP is also confirmed by lower values of $RI_{Std}$.

The use of k-fold cross-validation analysis has been also discussed in [15] in terms of the index (3). The results of the assessment of the classifier robustness against the patterns’ randomization showed values close to 100%.

As a further result, a study has been performed to compare the performance of the adopted MLP model against that of three other standard machine learning techniques. The results are reported in Table 3, and include the models’ accuracy evaluated with the test patterns of the dataset.

The results demonstrate that the investigated techniques are comparable in terms of accuracy. However, considering its ease-of-implementation in the embedded device, the MLP model has been considered as the most convenient model to be deployed in the sensing node.

**Figure 5:**
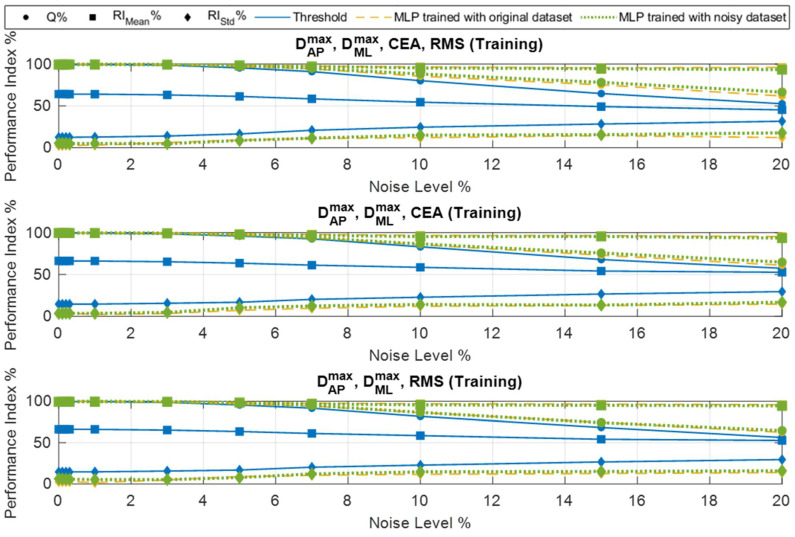

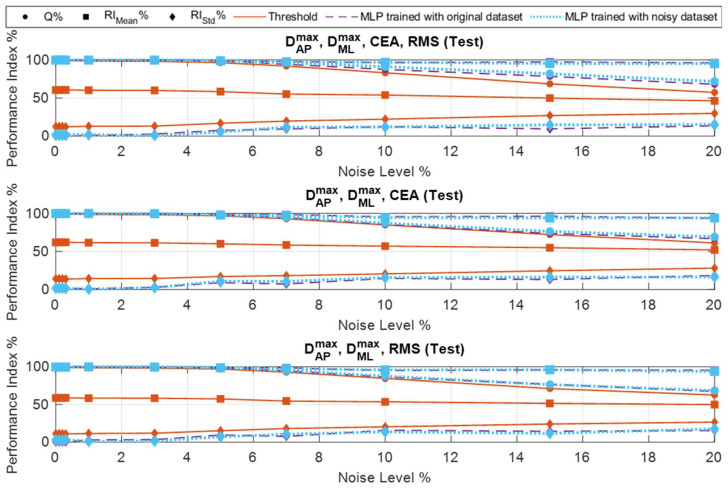
Performance of threshold and MLP algorithms as a function of noise levels in the input data, for both training and test subsets using different features.

As a first novel contribution of this work, the behavior of the classification model in the case of noisy input data was investigated. The task of this study is to assess the system’s robustness against noisy data, which could resemble the effects of exogenous dynamics (e.g., possible tremors or involuntary movements) or could be introduced by the electronics that are used (including sensors). For this purpose, two different studies have been performed. The first study aimed to investigate the behavior of the model trained by the original dataset, while a noisy dataset was used during the inference phase. To accomplish this task, the original dataset was corrupted with different levels of gaussian noise and, with the aim of uniformly dirtying the dataset, the standard deviation of the noise for each feature was fixed as a percentage of its maximum absolute values. In particular, the following noise levels were used: 0.1%, 0.2%, 0.3%, 1.0%, 3.0%, 5.0%, 7.0%, 10.0%, 15.0%, and 20.0%. For each noise level, the corrupted data were used to feed both threshold and MLP models, while indexes (3), (5), and (6) were estimated based on the models’ predictions. The results of this analysis are shown in Figure 5 for both the training and test patterns during the inference phase.

The second study investigated the convenience of also using noisy data to train the MLP model. To this end, a new training dataset called “Noisy dataset” was generated by merging the original training dataset (without noise) with the same data corrupted by five different levels (0.1%, 0.3%, 1.0%, 3.0%, and 5.0%) of noise. The results of this study are also shown in Figure 5.

As a further investigation, the performances of the classification models were evaluated using both all four features described in Section 3.1 and reduced subsets of features. In particular, the above analyses were repeated using models with the following set of features [$D_{AP}^{max}$, $D_{ML}^{max},\;CEA]$ and [$D_{AP}^{max}$, $D_{ML}^{max},\;RMS]$, respectively. This choice is justified by the fact that $D_{AP}^{max}$ and $D_{ML}^{max}$ are considered mandatory features for the representation of the user’s postural behavior. To analyze the effects of using three features instead of four, the two remaining possible combinations were considered by including CEA and RMS. The results of the above assessments are reported in Figure 5.

As can be observed, reducing the number of features from four to three does not significantly affect the system’s performance, in terms of its accuracy and reliability. This result allows for reducing the computational demand of the algorithm to be deployed in the embedded architecture and can be considered a further positive outcome of the investigation carried out through this work.

Regarding the study related to the use of noisy data, the following considerations emerge for the MLP model trained with the original dataset:

– A drop in accuracy is experienced as the noise level increases; however, the MLP algorithm performs better than the threshold algorithm, maintaining values of accuracy around 80% for a noise level up to 15.0%, in contrast to the 65% obtained for the threshold-based approach;
– The threshold algorithm shows a $RI_{Mean}$ of 60% with clean data, which decreases to 50% for increasing levels of noise. The MLP shows a $RI_{Mean}$ of almost 100% with a negligible drop (less than 5%) even at 20.0% of noise; this result demonstrates the better robustness of the MLP approach with respect to the threshold-based algorithm;
– Similar considerations also apply for the $RI_{Std}$ index, which, in the case of MLP, spans from 0% to around 13% for noise levels up to 20.0%, while the threshold-based algorithm shows values ranging from 10% to 31%; even this result indicates the better performance of the MLP algorithm;
– The above considerations apply to both the training and test subsets, with similar trends being shown for all the cases that were considered.

Finally, the MLP model trained with the noisy dataset, compared to the model trained with the original dataset, shows slightly higher accuracy values (increment of 5%) above a 10.0% noise level. Even in this case, high levels of the $RI_{Mean}$ index (with a minimum value above 93%) are observed.

## 5. Implementation of the MLP in the Embedded System: A Mixed-Postural Dynamics Test

In this section, the behavior of the machine learning algorithm deployed in the adopted embedded architecture is assessed by a dedicated test. The main purpose of this test is to validate the model when it is deployed in the device. Such validation was carried out using the same approach, setup, and environment already used during the development phase of the classification methodology. Future efforts will be dedicated to assessing the behavior of the system in different contexts, and will also involve end-users.

For this purpose, the MLP model was converted into C code and deployed using the following tools: STM32CubeIDE v1.11.2, STM32CubeProgrammer v2.14.0, and STM32CubeMX v6.7.0 with the X-CUBE-AI v8.0.1 expansion pack.

In particular, the X-CUBE-AI expansion pack, integrated in STM32CubeMX, enables the upload of Keras models saved in HDF5 format, with support for compression, optimization (balanced, inference time, RAM), validation using random or imported input/output data, and quantization. The above tools allowed for generating the C code of the model and its integration in a pre-existing firmware of the embedded device. For the specific case addressed in this work, the model was generated without any compression, and a balanced optimization was selected.

An experimental survey was carried out to compare the signal processing performed by the embedded sensing system with the same routine implemented in MATLAB^®^ R2024a. Specifically, a dedicated script was implemented to accomplish the following operations: importing the accelerometer raw data recorded by the sensing node; splitting the data into 10 s sliding windows with a 1 s shift; calculating, for each observation window, a full set of the four features considered in this work; implementing the MLP model imported via the Deep Learning Toolbox v24.1.

For the test, the following sequence of postural dynamics was reproduced by using the sensing node: ST, AP, UNST, ML, and again ST.

**Figure 6:**
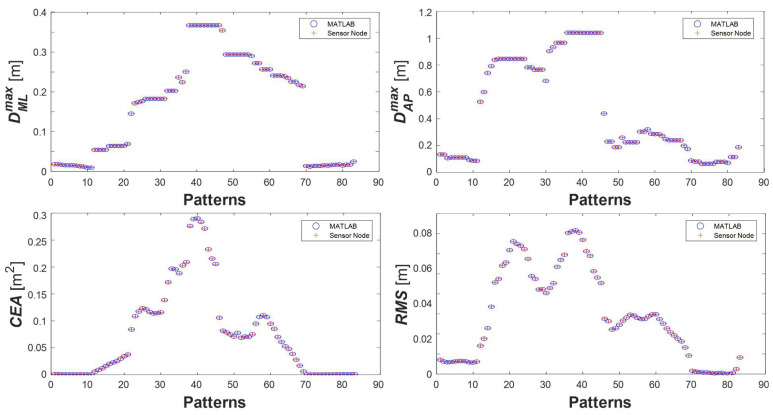
Results obtained by the experimental test to assess features estimated by the sensor node (+) against features estimated in MATLAB^®^ (o).

First, a comparison between the features generated by the sensing node and the features calculated in MATLAB^®^ was conducted. The result of this test is shown in Figure 6, which demonstrates suitable matching between the two sets of estimations.

**Figure 7:**
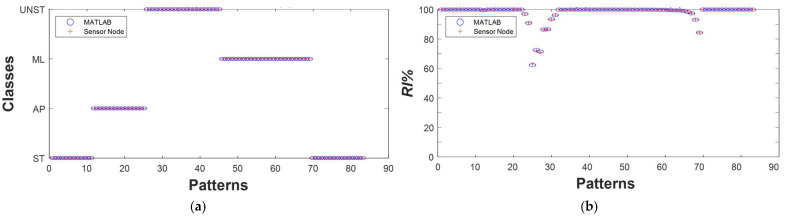
Results of a real-time test consisting of a sequence of postural dynamics. (**a**) Classes estimated by the sensor node (+) and predicted by the same algorithm running in MATLAB^®^ (o); (**b**) *RI* estimated by the sensor node (+) and by the MATLAB^®^ routine (o).

The second test was conducted to compare outcomes of the classification task performed by the sensing node and obtained in MATLAB^®^. As shown in Figure 7a, the two systems generate overlapping outputs that correspond to the expected postural dynamics.

The same applies for the *RI* reported in Figure 7b, for which the values are close to 100% in most cases, decreasing, as expected, during transitions between different postural dynamics.

## 6. Conclusions

The main aim of this work is to assess the performance of a machine learning approach for the classification of different classes of postural sway.

The achieved results demonstrate better performance of the MLP-based classification tool compared to threshold-based algorithms, in terms of both key accuracy and reliability performance indexes.

The suitability of using different sets of input features was also analyzed. The obtained results allow us to affirm that reducing the number of features to three does not significantly affect the performance of the classification algorithm, while this could be convenient to reduce the computational power required by the embedded system.

As a further analysis, the robustness of the classification algorithm against noise-corrupted data was investigated, which showed reliability index values above 95% in the case of datasets corrupted up to a 20.0% noise level. Also, training the MLP with a noisy dataset demonstrated benefits in terms of accuracy, especially when high noise levels were present, with the model still maintaining high reliability index values (above 93%). Overall, this can be considered the optimal choice among the different cases presented through this work, as it shows a good compromise between accuracy and reliability.

Moreover, the MLP model has been successfully deployed into the SensorTile.box embedded device. For the sake of assessment, a mixed-postural dynamics test was carried out, which allowed for a comparison between the system output and the results provided by the same MLP model running in MATLAB^®^.

The approach adopted through this paper is often used during the development phase of assistive technology tools. It is worth noting that collecting an appropriate amount of data by involving end-users in experimental trials might be incredibly time-consuming and complex. Moreover, this might be quite invasive, especially when the involvement of frail users is required. For this reason, during the early development phase, available datasets or emulating systems are often used, in order to perform a preliminary assessment of the methodology.

The use of an emulating architecture, such as the one adopted through this work, rather than using an already available dataset, might be a convenient strategy, since, in this case, data are generated by using the same hardware solution that will then be used in real application contexts. Furthermore, the model developed through the use of an emulated dataset can be successfully updated and improved by including patterns obtained from real users and using approaches such as transfer learning.

Future efforts will be dedicated to experimental surveys that involve real end-users. If required, a new training phase of the classification model will be performed, with the aim of considering peculiarities of specific exogenous signals (such as breathing or heartbeats) that come from operations with real users. Furthermore, another task will be the classification of different types of tremors in data collected from real patients. The development of a customized sensing platform that better fits users’ needs will be also be considered as a further advancement of this research activity.

## Figures and Tables

**Table 1:** Threshold-based and machine learning approaches for posture analysis and postural sway classification.

Target	Approach	
	Threshold-Based	Machine Learning-Based
Posture analysis	[19,21,23,24,25,26], [14,27,28,29] *	[30,31,35,36,37,38,39], [14] *
Classification of postural sway behaviors	[16,20,22]	[15], This work

* Works adopting frequency-based features; others mainly exploit time-based features.

**Table 2:** Performance comparison of threshold-based and MLP classification algorithms [15].

Data\Index	*Q*%	$RI_{Mean}\%$	$RI_{Std}\%$
Threshold algorithm			
Training	99.60	64.10	12.60
Test	99.10	60.50	12.50
MLP			
Training	99.90	99.79	3.54
Test	100.00	99.99	0.00

**Table 3:** Performance comparison with standard machine learning models.

Model	Test *Q*%
MLP	100.00
Random Forest Classifier	99.68
SVM	99.46
K-NN	99.36

## Data Availability

The data presented in this study are openly available in FigShare at https://doi.org/10.6084/m9.figshare.28847261.v1, accessed on 8 July 2025.

## Abbreviations

The following abbreviations are used in this manuscript:

PD	Parkinson’s Disease
CoP	Center of Pressure
MLP	Multi-Layer Perceptron
IMU	Inertial Measurement Unit
DWT	Discrete Wavelet Transform
NF	Neuro-Fuzzy
MCU	MicroController Unit
AP	Antero-Posterior
ML	Medio-Lateral
